# Assessing dose–response relationships for endocrine disrupting chemicals (EDCs): a focus on non-monotonicity

**DOI:** 10.1186/s12940-015-0029-4

**Published:** 2015-05-15

**Authors:** R Thomas Zoeller, Laura N Vandenberg

**Affiliations:** University of Massachusetts Amherst, Amherst, MA USA

**Keywords:** Non-monotonic, Risk assessment, Endocrine disruptor, Klimisch score, Good laboratory practices

## Abstract

The fundamental principle in regulatory toxicology is that all chemicals are toxic and that the severity of effect is proportional to the exposure level. An ancillary assumption is that there are no effects at exposures below the lowest observed adverse effect level (LOAEL), either because no effects exist or because they are not statistically resolvable, implying that they would not be adverse. Chemicals that interfere with hormones violate these principles in two important ways: dose–response relationships can be non-monotonic, which have been reported in hundreds of studies of endocrine disrupting chemicals (EDCs); and effects are often observed below the LOAEL, including all environmental epidemiological studies examining EDCs. In recognition of the importance of this issue, Lagarde et al. have published the first proposal to qualitatively assess non-monotonic dose response (NMDR) relationships for use in risk assessments. Their proposal represents a significant step forward in the evaluation of complex datasets for use in risk assessments. Here, we comment on three elements of the Lagarde proposal that we feel need to be assessed more critically and present our arguments: 1) the use of Klimisch scores to evaluate study quality, 2) the concept of evaluating study quality without topical experts’ knowledge and opinions, and 3) the requirement of establishing the biological plausibility of an NMDR before consideration for use in risk assessment. We present evidence-based logical arguments that 1) the use of the Klimisch score should be abandoned for assessing study quality; 2) evaluating study quality requires experts in the specific field; and 3) an understanding of mechanisms should not be required to accept observable, statistically valid phenomena. It is our hope to contribute to the important and ongoing debate about the impact of NMDRs on risk assessment with positive suggestions.

## Background

Paracelsus, considered the father of modern toxicology, stated, “All things are poison and nothing (is) without poison. Solely the dose determines that a thing is not a poison” [1]. This central dogma in toxicology is often re-stated as “the dose makes the poison”, which is not exactly the same, and has been taken to mean that the adverse effect of a toxin is proportional to the dose. The further assumption of a monotonic, if not linear, relationship between dose and effect is used as the foundation for modern risk assessments, where the effects of high doses are used to predict effects – and lack of effects – at lower doses.

In contrast, a group of independent scientists published in 2012 the first comprehensive review of the endocrine disrupting chemical (EDC) literature that revealed a large number of non-monotonic dose responses (NMDRs) in biochemical, animal and human studies [2]. The large number of NMDRs assessed led several groups to conclude that these dose responses are common for both hormones and EDCs [3-6]. Although non-monotonicity has received significant attention in the last few years, these phenomena are not new and their importance to risk assessment has been considered previously [7].

The 2012 literature review also spawned an intense debate about the reality of NMDRs and their importance for risk assessments (e.g., [8-11]). Some authors argued that because NMDRs are common for hormones, and for drugs that interact with hormone receptors, it is reasonable to predict that environmental chemicals that interact with hormone system would also exhibit NMDRs [2-4,6,12,13]. However, others argued that the data were insufficient to conclude that NMDRs are real or important (e.g., [10]). In general, this kind of debate is healthy and can provide the driving force for new science and new analyses. However, debate surrounding a controversy often paralyzes the risk assessment process. Therefore, a proposal to assess NMDRs using systematic criteria is important to bring this debate within the risk assessment domain.

### A new systematic approach to assess NMDRs

Considering the scientific climate and desire to develop approaches for the assessment of NMDRs, Lagarde et al. (2015) [14] published the first formal strategy for considering the use of datasets with NMDRs for inclusion in risk assessment. They propose a five step decision tree for the evaluation of NMDRs for their use in risk assessments: 1) The assessment of study quality; 2) determination of number of doses; 3) characterization of data for specific statistical analyses; 4) statistical analysis using defined criteria; and 5) assessment of biological plausibility.

The contribution made by Lagarde and colleagues is a significant advancement for the field of risk assessment, which was built on the expectation of monotonic dose responses. In this way, the Lagarde decision tree provides the first contribution by which NMDRs could be assessed and then used to identify ‘safe’ levels of chemical exposures.

However, from the perspective of basic science, we would like to address three elements of this decision tree: 1) the use of Klimisch scores to evaluate study quality, 2) the concept of evaluating study quality without topical experts’ opinions, and 3) the requirement of establishing the biological plausibility of an NMDR before consideration for use in risk assessment.

#### Klimisch scores

The first step in the Lagarde decision tree characterizes the quality of the study under consideration. The assessment of study quality is a standard step in the risk assessment process. In the study of EDCs, endocrinologists and environmental health scientists have proposed a series of criteria that should be met to consider a study “high quality” including the use of appropriate negative and positive controls, the use of sensitive animal species and strains, and the use of appropriate endpoints [3,15-18]. All of these criteria specifically focus on aspects of study design and are derived from an understanding of endocrine systems and their role in development and physiological control. In contrast, Klimisch et al. [19] propose a system for evaluating the quality of a scientific study based on its adherence to test guidelines and study reporting criteria including the employment of Good Laboratory Practices (GLP). Using the Klimisch scoring system, studies given the highest ranking (“Reliable without Restriction”) are those “…studies or data from the literature or reports [presumably not published in the peer review literature] which were carried out or generated according to generally valid and/or internationally accepted testing guidelines (preferably performed according to GLP) or in which the test parameters documented are based on a specific (national) testing guideline (preferably performed according to GLP) or in which all parameters described are closely related/comparable to a guideline method.” [19]. Importantly, as noted elsewhere, GLP criteria are typically only followed by industry-funded or government laboratories as these high-cost, personnel-intensive standards were developed in response to several examples of fraud committed in industry labs [20-22]; thus, the Klimisch score is an industry-developed method which typically gives the highest quality rankings to industry-funded studies [23].

Unfortunately, the Klimisch scoring system confounds quality in study design and execution (which are directly related to quality in the resulting data) with quality in recordkeeping and study reporting. For example, GLP compliant studies do not prevent test substance contamination of the untreated control group [24], or guarantee that the positive control group has responded as expected [17], or that the tissues being studied have been dissected appropriately [21]. Other groups have similarly noted that the conflation of quality of reporting and quality of data is problematic [25].

There are also significant weaknesses in guideline studies as they relate to EDCs – whether or not they are performed according to GLP [4,20]. For example, most guideline studies only examine three treatment doses, which is not sufficient to make a conclusive judgment about the shape of the dose response curve. Similarly, guideline studies can be performed on animal species and strains that are insensitive to hormones, and thus are not appropriately responsive to EDCs at low doses [26]. Moreover, the endpoints assessed in traditional guideline studies do not address the most important chronic diseases in human populations today (e.g., [17]) and therefore will limit the utility of the overall risk assessment process. Unfortunately, the use of Klimisch scores will restrict the endpoints considered “adverse” to those endpoints captured by traditional guideline studies. Thus, while a focus on study quality is a positive aspect of the Lagarde proposal, the reliance on Klimisch scores is a major weakness.

#### Use of topical experts

Klimisch scores are more of a bureaucratic strategy than a scientific one, and provide a rationale allowing for non-experts to evaluate study quality with confidence, even when they do not understand the underlying biology of the study at hand. The engagement of topical experts would clearly produce difficult challenges, in part because the examples of NMDRs in EDC studies occur at many levels of investigation (e.g., *in vitro*, animal study, human study), on many different hormone systems (of which there are 10 or so that are evaluated as targets of chemical actions), and at multiple life cycle stages. The complexity of these issues should not be underestimated. As an example from our experience, thyroid hormone has very specific effects during brain development (i.e. processes that occur *in utero* and the early postnatal period). In rodent studies of brain development, thyroid hormone regulates the expression of different genes through different receptors, in a temporally and spatially specific manner. Studies in genetic strains of mice have revealed which isoform of thyroid hormone receptor is responsible for certain features of thyroid hormone action on brain development [27]. Likewise, painstaking studies over development demonstrate that a single gene (e.g., RC3) is regulated by thyroid hormone in some – but not all – regions of the brain, and at some – but not all – times during development [28].

The complexity of the thyroid system and methods for studying it led to the development of an 81-page document by the American Thyroid Association [29] to guide investigators in this domain and improve the overall quality of research in this area. This complexity obviously extends to other hormones, physiological processes and organisms, including humans. Therefore, to evaluate the quality of a study designed to inform us about the ability of a manufactured chemical to interfere with hormone action, experts in the hormone system and physiological events under study must be recruited to contribute to this essential exercise. The Lagarde et al. method [14] does not specifically address the importance of specialists in the endpoint of interest when risk assessments are being conducted, but their use of the Klimisch score should again be reconsidered for this reason. It is also important to recognize that the strategy of Klimisch et al. is to improve the overall quality of the science being considered in a regulatory decision by excluding studies about which there is some question. We propose that an alternate strategy would improve risk assessment in general: to identify the strength of each study – as well as their limitations – and determine the role of that information in hazard identification and characterization. This will require specialists.

#### Biological plausibility of NMDRs

The Lagarde et al. proposal first includes a rigorous evaluation of the statistical validity of the NMDR under study. This strategy will eliminate simple outliers in datasets, or when the dataset does not include enough dose levels to reasonably determine the shape (monotonicity versus non-monotonicity) of the dose–response relationship. This is an important issue because datasets can be quite complex and even guideline studies can be filled with random fluctuations. However, the second phase focuses on evaluating the biological plausibility of the dose response relationship. That is, what is the mechanism that produces this dose–response curve? There are two weaknesses with this concept. First, understanding mechanisms that link specific chemical exposures to specific outcomes is highly complex and time consuming, even though several general mechanisms by which NMDRs can be produced are known (e.g. [16,30-32]). In many cases, understanding the mechanism underlying a dose–response shape could take years, or decades, after the discovery of a biological phenomenon. For example, the mechanism (s) by which polychlorinated biphenyls (PCBs) produce neurotoxicity can still be debated nearly 40 years after their production was banned, whereas the phenomenon itself (neurotoxicity) is widely acknowledged [33,34]. Moreover, non-specialists will make the judgment regarding biological plausibility of a dose response, providing an enormous opening for variability in the application of this decision tree, and one potentially driven by agendas. Second, the fact that this mechanistic determination is being required of a chemical exhibiting an NMDR with some health outcome is in contrast to chemicals that produce a monotonic dose–response. Monotonic dose response phenomena are accepted as the default in risk assessments, even when the mechanism is not understood. Thus, there is an inherent asymmetry in the analysis, which reveals a fundamental bias in the approach. In this way, the Lagarde decision tree creates a situation where it is possible for a statistically valid NMDR concerning important adverse effects to be ignored if a risk assessor “feels” that the biological mechanism for the observed non-monotonicity is not sufficiently well understood. These issues should be addressed before it is used in the risk assessment process. Specifically, mechanisms should not be required to accept biological observations or phenomena in the risk assessment process, and non-monotonic and monotonic dose responses should be treated equally in these assessments.

## Conclusions

The “risk-based approach” to chemical safety is balanced on the principle that all chemicals are toxic, that ‘the dose makes the poison’, and that there are no adverse effects below the calculated “safe” level. If, and only if, these principles are true, the human population can be safely exposed to hundreds of toxic chemicals simultaneously as long as the exposure to each one is below the level calculated with these assumptions. The risk assessment process in general has been challenged for EDCs, and one part of this challenge is the inability of this risk-based approach to adapt to NMDRs, which are common for this class of chemicals. Recently, several academic and government groups have developed methods to improve the processes of systematic review [25,35,36]. The Lagarde decision tree provides methods by which NMDRs can be assessed and included in a risk assessment. This is important because dose response data are combined with hazard assessment and exposure data in the risk characterization process; if a non-monotonic relationship is apparent between chemical dose and an adverse outcome, extrapolation from high doses that are ‘toxic’ to lower doses, presumed to be safe, should not be done [2,3]. We have reviewed three areas in which the Lagarde decision tree should be improved. With these relatively minor, but very important amendments, this decision tree could offer vast progress for the risk assessment community.

## Abbreviations

EDCs: Endocrine disrupting chemicals
EPA: Environmental protection agency
GLP: Good laboratory practice
LOAEL: Lowest observed adverse effect level
PCBs: Polychlorinated biphenyls
